# Primary and metastatic ovarian cancer: Characterization by 3.0T diffusion-weighted MRI

**DOI:** 10.1007/s00330-017-4786-z

**Published:** 2017-03-13

**Authors:** Auni Lindgren, Maarit Anttila, Suvi Rautiainen, Otso Arponen, Annukka Kivelä, Petri Mäkinen, Kirsi Härmä, Kirsi Hämäläinen, Veli-Matti Kosma, Seppo Ylä-Herttuala, Ritva Vanninen, Hanna Sallinen

**Affiliations:** 1 Department of Gynaecology and Obstetrics, Kuopio University Hospital, Kuopio, Finland; 2 Institute of Clinical Medicine, School of Medicine, Gynaecology, University of Eastern Finland, Kuopio, Finland; 3 Department of Clinical Radiology, Kuopio University Hospital, Kuopio, Finland; 4 Department of Biotechnology and Molecular Medicine, A.I. Virtanen Institute for Molecular Sciences, University of Eastern Finland, Kuopio, Finland; 5 Department of Pathology and Forensic Medicine, Kuopio University Hospital, Kuopio, Finland; 6 Institute of Clinical Medicine, School of Medicine, Pathology and Forensic Medicine, University of Eastern Finland, Kuopio, Finland; 7 Cancer Center of Eastern Finland, University of Eastern Finland, Kuopio, Finland; 8 Institute of Clinical Medicine, School of Medicine, Clinical Radiology, University of Eastern Finland, Kuopio, Finland

**Keywords:** Ovarian neoplasms, Neoplasm metastasis, Neovascularization pathologic, Cell proliferation, Diffusion magnetic resonance imaging

## Abstract

**Objectives:**

We aimed to investigate whether apparent diffusion coefficients (ADCs) measured by 3.0T diffusion-weighted magnetic resonance imaging (DWI) associate with histological aggressiveness of ovarian cancer (OC) or predict the clinical outcome. This prospective study enrolled 40 patients with primary OC, treated 2011-2014.

**Methods:**

DWI was performed prior to surgery. Two observers used whole lesion single plane region of interest (WLsp-ROI) and five small ROIs (S-ROI) to analyze ADCs. Samples from tumours and metastases were collected during surgery. Immunohistochemistry and quantitative reverse transcription polymerase chain reaction (qRT-PCR) were used to measure the expression of vascular endothelial growth factor (VEGF) and its receptors.

**Results:**

The interobserver reliability of ADC measurements was excellent for primary tumours ICC 0.912 (WLsp-ROI). Low ADCs significantly associated with poorly differentiated OC (WLsp-ROI *P* = 0.035). In primary tumours, lower ADCs significantly associated with high Ki-67 (*P* = 0.001) and low VEGF (*P* = 0.001) expression. In metastases, lower ADCs (WLsp-ROI) significantly correlated with low VEGF receptors mRNA levels. ADCs had predictive value; 3-year overall survival was poorer in patients with lower ADCs (WLsp-ROI *P* = 0.023, S-ROI *P* = 0.038).

**Conclusion:**

Reduced ADCs are associated with histological severity and worse outcome in OC. ADCs measured with WLsp-ROI may serve as a prognostic biomarker of OC.

***Key Points*:**

• *Reduced ADCs correlate with prognostic markers: poor differentiation and high Ki-67 expression*

• *ADCs also significantly correlated with VEGF protein expression in primary tumours*

• *Lower ADC values are associated with poorer survival in ovarian cancer*

• *Whole lesion single plane-ROI ADCs may be used as a prognostic biomarker in OC*

## Introduction

Ovarian cancer is the fifth most frequent cancer among females and the fourth most common cause for female cancer mortality [1]. The treatment of ovarian cancer (OC) has developed rapidly during the last few decades, but the prognosis remains poor. Although OC is sensitive to chemotherapy, up to 70% of patients relapse during the first 3 years after the primary treatment [1]. Survival in OC is related to age at diagnosis, stage, histopathological grade and, most of all, size of residual tumour after sytoreductive surgery [2]. Neoadjuvant chemotherapy is an important additional treatment modality in cases where the tumours are widely spread and optimal surgical result is not possible without chemotherapy [3].

Molecular pathophysiology of OC is well documented and various biological markers have been reported to have prognostic significance. Ki-67 is a nuclear protein associated with cellular proliferation; higher Ki-67 expression in OC is associated with more aggressive disease and worse clinical outcome [4]. Angiogenic growth factors and their receptors promote and regulate angiogenesis which is essential in tumour progression. Tumours require neovascularization for growth. The vascular endothelial growth factor (VEGF) family is the most studied: VEGFs have mitogenic, angiogenic, and vascular hyperpermeability effects on tumours [1, 5–8]. VEGF, -B, -C, and -D signal through three tyrosine kinase receptors: VEGFR-1 (Flt-1), VEGFR-2 (KDR/Flt-1), and VEGFR-3 (Flt-4) [5].

Diffusion-weighted imaging (DWI) and assessment of apparent diffusion coefficients (ADCs) have recently been introduced as new tools in abdominal imaging and may help to improve assessment of the metastatic spread of OC at the time of diagnosis and during follow-up [9–14]. ADCs are affected by tissue cellularity, fluid viscosity, membrane permeability, macromolecular structures, and blood flow [15]. Due to high cellularity, malignancies are associated with lower ADCs [11, 12, 16–19]. However, no standardized measurement protocols or cut-off values are available for ADC measurements in OC. The scanner type and size and positioning of regions of interest (ROI), and most importantly b-values have varied between studies, affecting the differences in ADC values. The purpose of the present study was to investigate whether ADCs measured by 3.0T DWI are associated with histological severity in OC or predict the clinical outcome in patients with OC.

## Materials and methods

### Patients and study design

This was a prospective single-institution study at Kuopio University Hospital between 2011 and 2014. The Research Ethical Committee approved the study protocol. Written informed consent was obtained from all patients prior to enrollment. A total of 40 patients with primary OC (mean age 66 years, range 47-86) treated at Kuopio University Hospital were included in this study. Patients were followed up until June 2016. The eligibility criteria were: clinical diagnosis of primary OC, fallopian tube cancer, or peritoneal carcinoma; measureable disease at staging computer tomography (CT); and no contraindication to MRI. Cancer staging was based on the standards of the International Federation of Gynecology and Obstetrics (FIGO). Histological type and grade were evaluated according to the World Health Organization (WHO) criteria. All patients underwent diagnostic 3.0T MRI before any treatment with a structured protocol including DWI. Four patients were excluded from imaging analyses because of severe artifacts: sterilization clip-on (*n* = 1), motion artifact (*n* = 2), and hip prosthesis (*n* = 1) that strongly degraded the image.

Samples from tumours and metastases for immunohistochemistry and quantitative reverse transcription polymerase chain reaction (qRT-PCR) analyses were collected during surgery. Five patients receiving neoadjuvant chemotherapy were excluded from histopathological and qRT-PCR analyses because chemotherapy causes cellular damage to tumour cells. The patients received paclitaxel-carboplatin as adjuvant chemotherapy after an operation, excluding one stage 1A patient with single carboplatin. Twelve patients received also bevacizumab either in the primary setting (*n* = 8) if disease was stage IIIC-IV and there was residual tumour, or in a recurrent situation (*n* = 4), if they had not received it earlier. The decision to give bevacizumab for high-risk patients was based on the protocol that was used in the ICON-7 trial [20]. The patient characteristics are described in Table 1.

**Table 1:** Clinicopathological characteristics of patients with ovarian cancer (*N* = 40) and the mean apparent diffusion coefficient (ADC) values of the primary tumours in corresponding subgroups of patients

Variable	*n* (%)	Mean ADC^a^	*P*
Ascites	29 (73)	0.820	0.432
No ascites	11 (28)	0.882	
BMI > 25 kg/m^2^	23 (58)	0.803	0.297
BMI ≤ 25 kg/m^2^	16 (40)	0.900	
CA12-5 ≤ 403	21 (53)	0.868	0.350
CA12-5 > 403	19 (48)	0.798	
Histological grade			0.035
1	2 (5)	1.232	
2	13 (33)	0.864	
3	25 (63)	0.784	
Stage at diagnosis			0.079
I	5 (13)	0.942	
II	2 (5)	1.192	
III	17 (43)	0.760	
IV	16 (40)	0.852	
Histological type			0.637
Serous high grade	28 (70)	0.801	
Endometrioid	5 (13)	0.931	
Mucinous	2 (5)	1.013	
Clear cell	1 (2)	0.783	
Other	4 (10)	0.842	
Primary residual tumour			0.232
None	16 (40)	0.896	
≤1 cm	17 (42.5)	0.817	
>1 cm	7 (17.5)	0.753	
Chemotherapy response			0.433
Neoadjuvant	5 (12.5)		
Complete response	30 (75)	0.857	
Partial response	3 (7.5)	0.727	
Stable disease			
Progressive disease	7 (17.5)	0.802	
Tumour recurrence			0.723
No recurrence	22 (55)	0.851	
Recurrence	18 (45)	0.802	
Patient status			
Dead, ovarian cancer	16 (40)		
Alive	24 (65)		

ADC = apparent diffusion coefficient, BMI = body mass index

^a^Mean value × 10^-3^ mm^2^/s when using the whole lesion single plane covered region of interest

### Imaging protocol and image analysis

MRI was performed with a 3.0T scanner (Philips Achieva 3.0T TX, Philips N.V., Eindhoven, The Netherlands) and a body coil (Sense-XL-Torso) covering the whole abdomen from the lower thorax to the symphysis. The protocol included transaxial, sagittal, and coronal T2-weighted (repetition time (TR) 651 ms, echo time (TE) 80 ms) and transaxial fat-suppressed spectral attenuated inversion recovery (SPAIR) and DUAL- fast field echo (FFE) sequences, and DWI (b-values 0, 300, 600 mm^2^/s) and DWI with body signal suppression (diffusion-weighted imaging with background body single suppression (DWIBS), b-value 800 mm^2^/s). A DWIBS sequence was used for visual detection of tumours. Breath hold was not used in the lower abdomen DWI_3b sequence, but was used in the upper abdomen, where breathing movements are more likely to affect the image quality. ADC maps were automatically generated for b-values of 0, 300 and 600 mm^2^/s. ADC data was fitted mono-exponentially by using these three b-values. The detailed imaging protocol is described in Table 2.

**Table 2:** Imaging protocol

Sequence acquisition time	Orientation	TR (ms)	TE (ms)	Flip angle (°)	FatSat	Resolution (mm)	*N* slices (gap mm)	SENSE factor	Other
Lower abdomen									
T2W_TSE *0:41.3*	tra	Shortest	80	90	-	0.7x0.7x5.0	52 (0.5)	2.0	Breath hold
T2W_TSE *0:35.9*	sag	Shortest	80	90	-	0.7x0.7x5.0	61 (0.5)	2.0	Breath hold
T2W_TSE *0:33.0*	cor	Shortest	80	90	-	0.7x0.7x5.0	58 (0.5)	2.0	Breath hold
DWIBS *3:35.7*	tra	Shortest	Shortest	-	-	1.3x1.3x5.0	62 (0)	2.0	b = 800
DWI_3b *3:40.6*	tra	Shortest	Shortest	-	STIR	1.8x1.8x5.0	56 (0.5)	2.0	b = 0, 300, 600
dual_FFE *1:13.4*	tra	180	1.15 (outphase) 2.30 (inphase)	55	-	1.3x1.3x5.0	56 (0.4)	2.0	Breath hold
Upper abdomen									
T2W_TSE *2:24.3*	tra	Shortest	80	90	-	0.7x0.7x5.0	48 (0.5)	2.0	Navigator
T2W_SPAIR *2:24.0*	tra	Shortest	70	90	SPAIR IR = 90 ms	0.7x0.7x5.0	48 (0.5)	2.0	Navigator
DWIBS *3:08.7*	tra	Shortest	Shortest	-	-	1.3x1.3x5.0	53 (0)	2.0	Navigator b = 800
DWI_3b *2:21.8*	tra	Shortest	48	-	STIR	1.7x1.7x5.0	48 (0.5)	2.0	Breath hold b = 0, 300, 600
dual_FFE *1:13.4*	tra	180	1.15 (outphase) 2.30 (inphase)	55	-	1.3x1.3x5.0	56 (0.4)	2.0	Breath hold
T1_FS *0:20.2*	tra	Shortest	Shortest	10	SPAIR IR=shortest	1.5x1.5x3.0	147 (0)	2	Breath hold

TR = repetition time, TE = echo time, FatSat = fat saturation, *N* slices = number of slices, tra = transversal, sag = sagittal, cor = coronal, TSE = turbo spin echo, DWIBS = diffusion-weighted imaging with background body signal suppression, SPAIR = spectral attenuated inversion recovery, FFE = fast field echo, FS = fat saturation, IR = inversion recovery

**Fig. 1:**
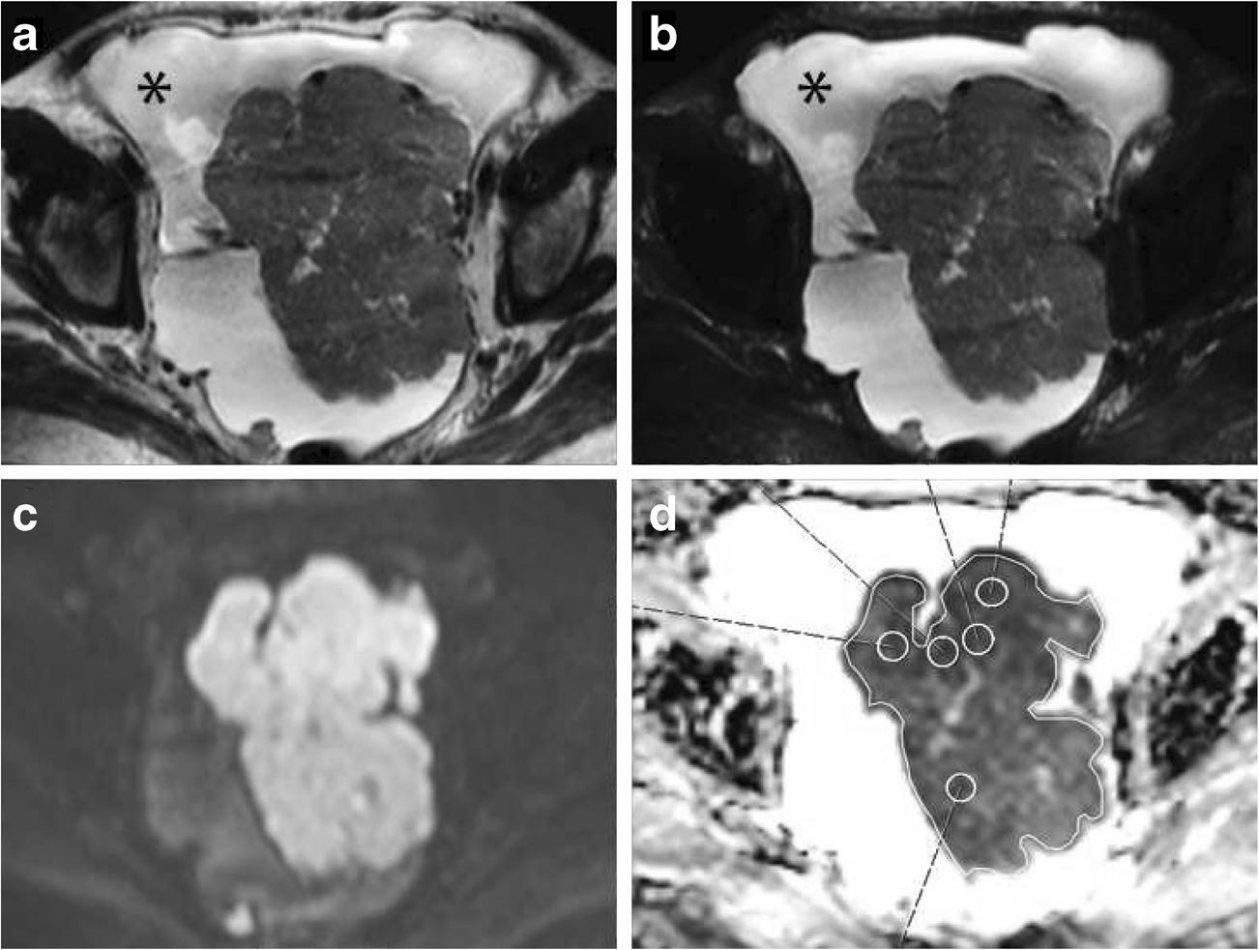
Images in a 67-year-old woman with high grade serous ovarian adenocarcinoma. A large primary tumour was imaged with (**a**) T2-weighted, (**b**) T2 spectral attenuated inversion recovery (SPAIR) fat-saturated, and (**c**) diffusion-weighted imaging with background body signal suppression (DWIBS) (b 800) MRI. * Bright ascites in (**a**) and (**b**). The tumour appears dark in the apparent diffusion coefficient (ADC) map (**d**), which illustrates the region of interest placement for ADC measurements. The whole lesion single plane region of interest (WLsp-ROI) was placed to cover the whole tumour in the slice in which the tumour appeared largest. ADC value is 0.695 × 10^-3^mm^2^/s. The five small ROIs (S-ROI) were placed on subregions appearing to have the lowest signal on the ADC map. Lowest ADC value 0.543 × 10^-3^mm^2^/s, is used in statistical analyses

Two observers (A.L, S.R, with 2 and 10 years of experience in gynecological imaging) independently and, blinded to histological information, evaluated all MRI data using a Sectra-PACS workstation (IDS7, Version15.1.20.2, Sectra AB, Linköping, Sweden). ADC values were measured from the whole lesion covering region of interest (WLsp-ROI) from the single plane where the tumour appeared largest and five small subregion ROIs (S-ROI, 1 cm in diameter) that were drawn both in the primary tumours and in omental cake or peritoneal lesions (Fig. 1). Cystic and necrotic areas were meticulously avoided, as they may erroneously increase ADCs. Small ROIs were placed on the subregions that were most bright in DWIBS images and had the lowest signal intensity in ADC maps. Throughout the text all ADC values are quoted with units of x10^-3^mm^2^/s.

### Immunohistochemistry

Tissue samples were embedded in paraffin and cut into 5-μm-thick sections. The sections were processed for hematoxylin-eosin, VEGF (Santa Cruz, 1:250), HIF-1α (Novus 1:75), Ki-67 (DAKO 1:100), Caspase-3 (Cell Signaling 1:500), CD34 (DAKO 1:500), CD105 (DAKO 1:90), and D2-40 (DAKO 1:200) staining. HIF-1α expression was analyzed in epithelial OC cells from the nucleus and cytoplasm. VEGF expression was evaluated in the epithelium and stroma. Ki-67 and Caspase-3 were analyzed in the nucleus. The percentage of stained cells was calculated.

The number of microvessels, mean microvessel area (μm^2^), microvessel density, and total microvascular area (%) in the tumours were measured from CD34-, CD105-, D2-40- immunostained sections using analySIS software at 200× magnification in a blinded manner. Three different fields representing maximum microvessel areas were selected from each tumour [21, 22]. Necrotic areas were avoided. Five patients were excluded from this analysis due to neoadjuvant chemotherapy.

### Quantitative RT-PCR

RNA was isolated using TRI-reagent (Sigma Aldrich). The cDNA was synthesized from 5 μg of total RNA using random hexamer primers (Promega) and RevertAid^TM^ reverse transcriptase (Fermentas) after treating the samples with DNase (Promega). The expression of mRNAs encoding VEGF, VEGF-C, VEGF-D, VEGFR-1, VEGFR-2, and VEGFR-3 was measured according to the manufacturer’s protocol (StepOnePlus, Applied Biosystems) using specific Assays-on-Demand target mixes (Applied Biosystems). The expression levels were normalized to peptidylprolyl isomerase A (PPIA), and the results are shown as relative expression. Five patients having neoadjuvant chemotherapy were excluded from this analysis.

### Statistical analysis

SPSS for Windows (Version 22.0, 1989-2013, SPSS Inc., Chicago, USA) was used for statistical analyses. ADCs from the WLsp-ROI and the lowest ADCs from the S-ROIs were used. Values are presented as mean ± SD unless otherwise stated. An interclass correlation coefficient (ICC) was used to test interobserver correlation in continuous variables. The Bland-Altman method was used to visualize interobserver variability. The Kruskal-Wallis test and the Mann Whitney U-test were used when appropriate. Bivariate correlations for continuous variables were analyzed using Spearman’s test. Wilcoxon signed rank test was used to compare ADCs, histology, and qRT-PCR results between primary ovarian lesions and related metastases. For the survival analyses, ADCs were dichotomized into low and high values using the median as a cut-off. The Kaplan-Meier method (log-rank) was used in univariate survival analyses. Significant variables from the univariate analyses were entered in a stepwise manner for Cox regression multivariate analysis. Overall survival (OS) was defined as the time interval between the date of surgery and the date of death or the end of follow-up. Recurrence-free survival (RFS) was defined as the interval between the date of surgery and the date of identified recurrence. *P* < 0.05 was considered significant, and high statistical significance was set at *P* < 0.01.

## Results

**Fig. 2:**
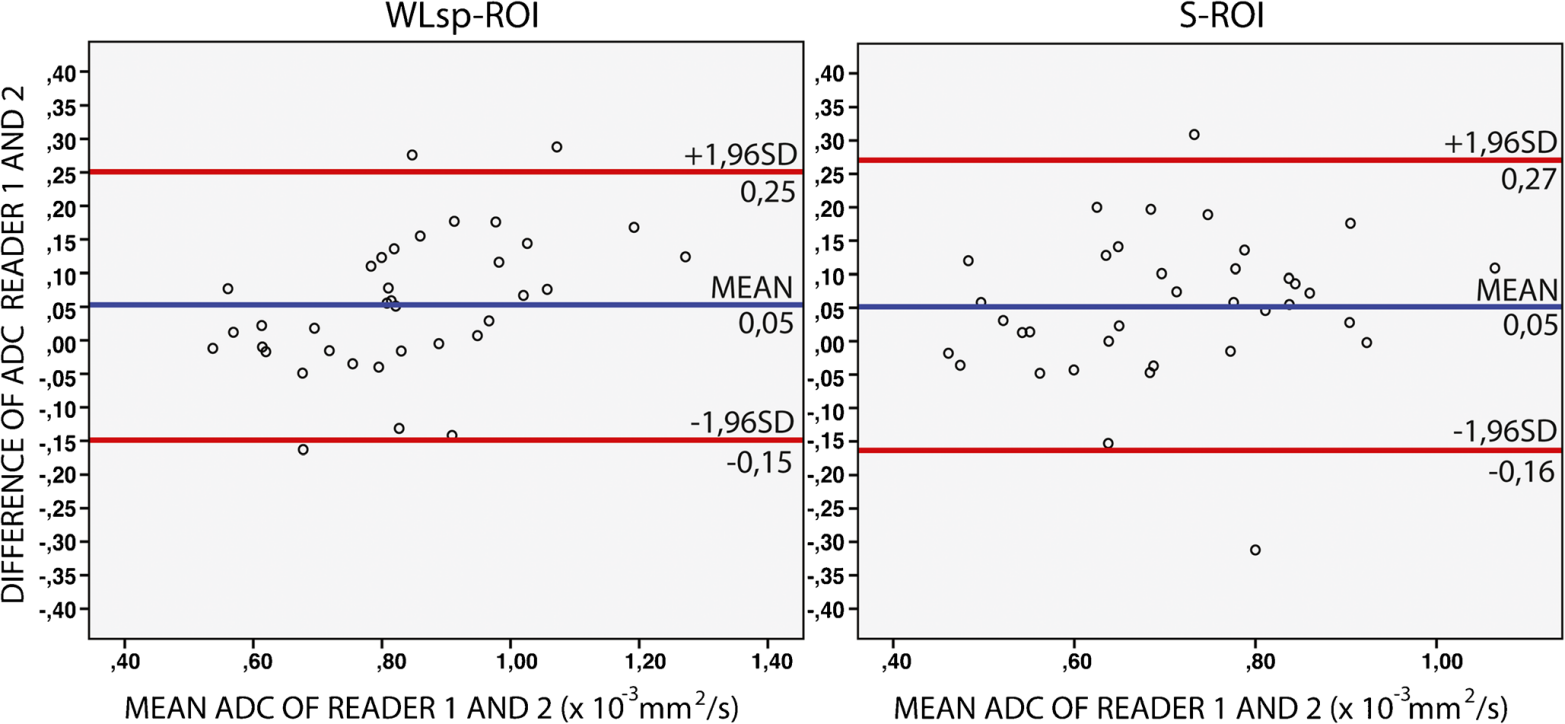
Bland Altman plots show ADC measurements in a whole lesion single plane region of interest (WLsp-ROI) and a small subregion region of interest (S-ROI) positioning as performed by the two readers. The difference in ADC values between two readers (y-axis) is plotted against the mean ADC of both readers (x-axis). The red line represent the mean absolute difference (bias) in ADC between the two readers; the blue lines represent the 95% confidence intervals (1.96 times the standard deviation) of the mean difference (limits of agreement). The mean absolute difference in ADC measurements between two readers is higher when using S-ROI

**Fig. 3:**
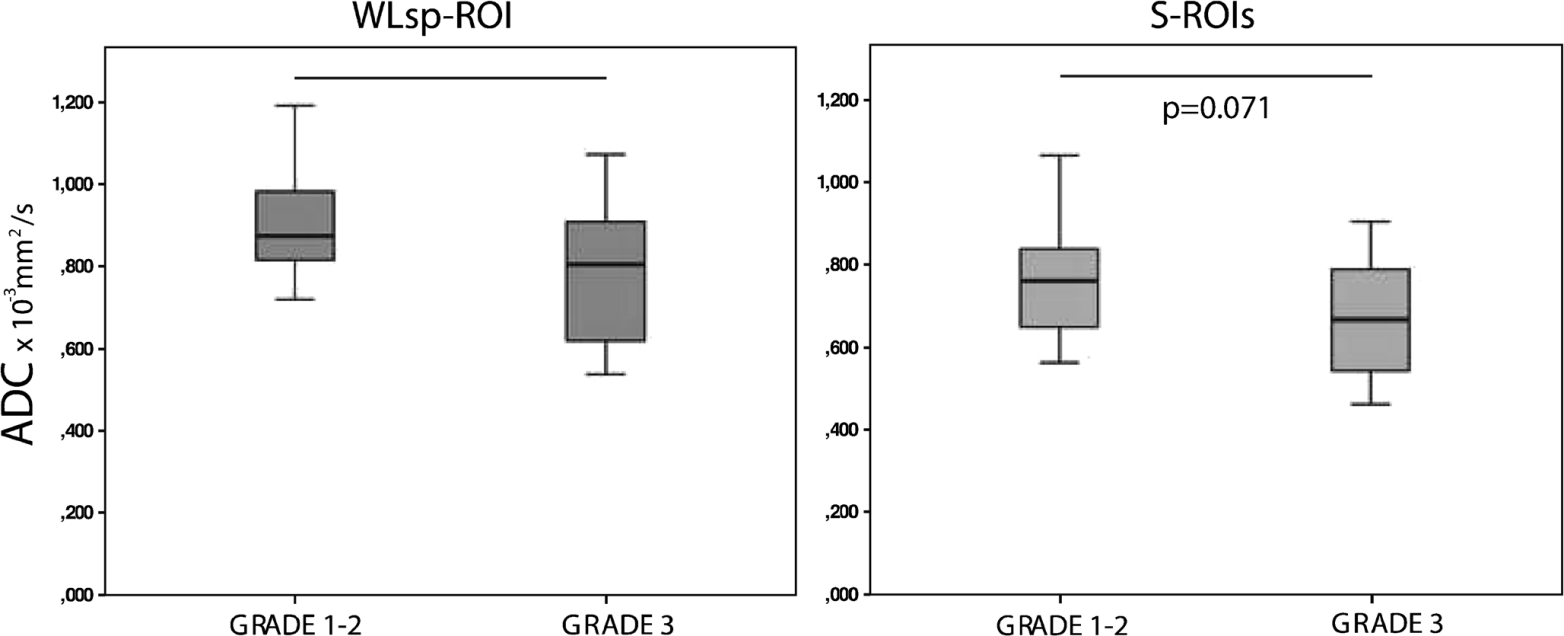
Relationship between apparent diffusion coefficients (ADCs) and the histopathological grade of ovarian cancer. Lower grade cancer was associated with significantly higher ADCs in the whole lesion covering region of interest (WLsp-ROI) (A) and in the small subregion regions of interest (S-ROI) (B) of the primary tumour. Whiskers represent standard deviation. ADCs measured from the WLsp-ROI were higher than those measured from the S-ROI (*P* < 0.001)

The mean largest diameter of a tumour in the plane where WLsp-ROI was placed was 77.6 mm (range 23-230 mm). The interobserver agreement of the ADC measurements was excellent for primary tumours (ICC 0.912 for WLsp-ROI, 0.856 for S-ROI). For metastatic lesions (*n* = 27) the agreement was good (ICC 0.705 for WLsp-ROI, 0.746 for S-ROI). The Bland-Altman method was used to visualize interobserver reproducibility (Fig. 2) The Bland-Altman 95% limits of agreement were -0.15 – 0.25 x10^-3^mm^2^/s for WLsp-ROI and -0.16 – 0.27 x10^-3^mm^2^/s for S-ROI and coefficients of reproducibility were 0.22 and 0.23, respectively. ADCs measured from WLsp-ROIs were significantly higher than those measured from S-ROIs. Lower ADCs were associated with poorly differentiated histology of grade 3 WLsp-ROI *P* = 0.035, S-ROI *P* = 0.071 (Fig. 3). Grade 1 (*n* = 2) and 2 (*n* = 13) were pooled together to achieve a statistically appropriate group size. There were no significant associations with age, tumour size, FIGO stage, ascites, ca12-5 level, parity, time of menopause, smoking, or obesity.

### Histopathological and qRT-PCR analyses

VEGF protein expression in epithelial cells was significantly higher in metastases than in related primary lesions (*P* = 0.008). Ki-67 correlated inversely with VEGF protein expression in primary tumours *r* = -0.717, *p* < 0.001. The mean size of lymphatic vessels (D2-40) was significantly larger in metastases (962.83 μm ± 794.24) than in primary lesions (565.36 μm ± 302.42, *P* = 0.019). There were no significant differences found in other histopathological analyses (HIF-1, Caspase-3, CD-34, CD105, D2-40).

**Fig. 4:**
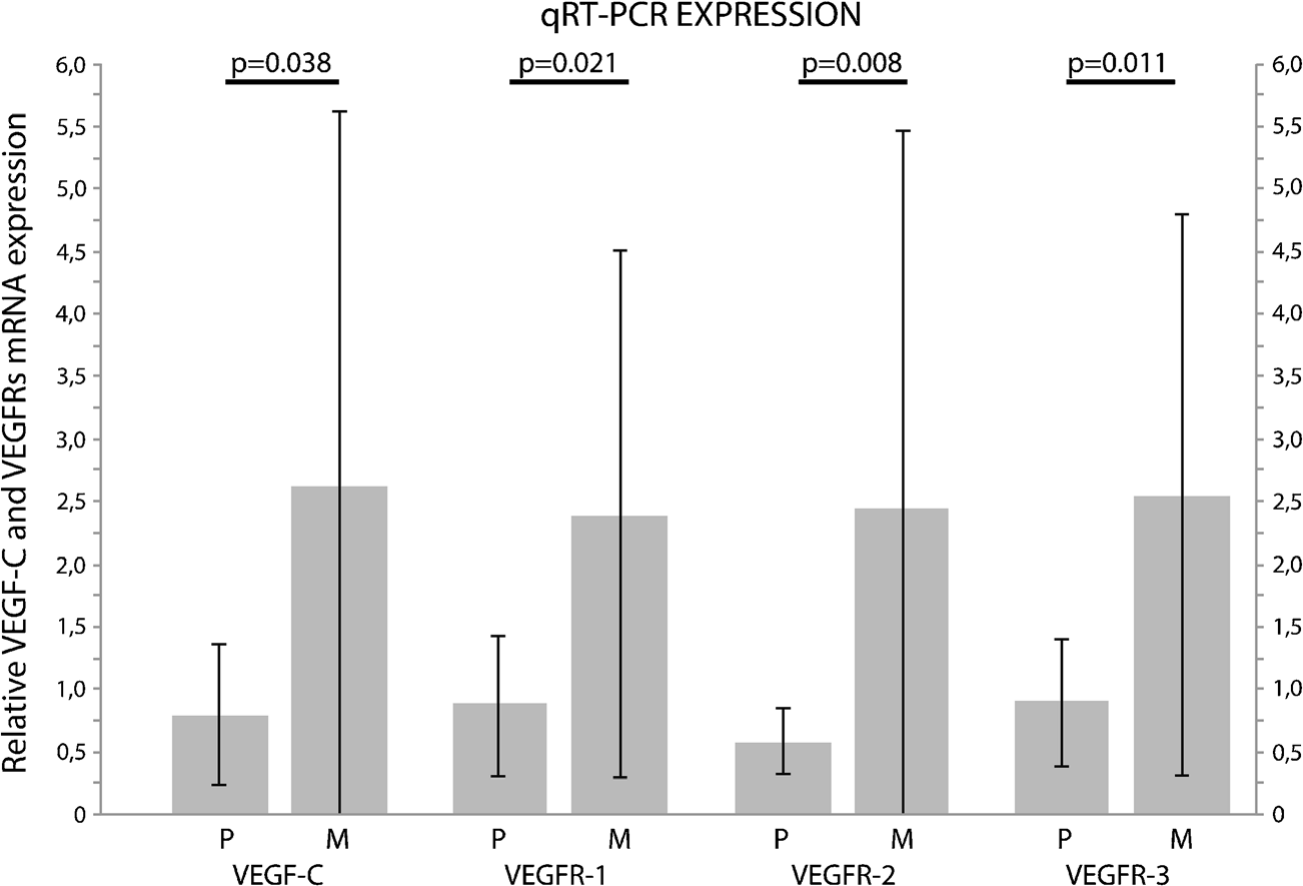
Differences in vascular endothelial growth factor C (VEGF-C) and VEGF receptors (VEGFR) mRNA levels in metastases and primary tumours (*n* = 35). VEGF-C (*P* = 0.038) and VEGFR-1 (*P* = 0.021), VEGFR-2 (*P* = 0.008), and VEGFR-3 (*P* = 0.011) relative expressions were higher in metastases (M) than in related primary lesions (P) according to quantitative reverse transcription polymerase chain reaction (qRT-PCR) analyses. Box-plots represent mean and whiskers standard deviation. The expression levels were normalized to peptidylprolyl isomerase A (PPIA)

The expression of VEGF-C mRNA was higher in metastases (2.63 ± 2.98) than in related primary lesions (0.79 ± 0.56, *P* = 0.038). VEGF and VEGF-D mRNA levels did not differ significantly between primary tumours and metastatic lesions (VEGF: 1.93 ± 3.31 vs. 1.298 ± 1.45, *P* = 0.859; VEGF-D: 5.38 ± 10.68 vs. 0.51 ± 0.54, *P* = 0.110). However, in all VEGFRs mRNA expression was higher in metastases than in related primary tumours (VEGFR-1: 2.39 ± 2.11 vs. 0.89 ± 0.56, *P* = 0.021; VEGFR-2: 2.44 ± 3.02 vs. 0.58 ± 0.26, *P* = 0.008; VEGFR-3: 2.54 ± 2.24 vs. 0.91 ± 0.51, *P* = 0.011; Fig. 4).

### Correlation between ADCs and histology

**Fig. 5:**
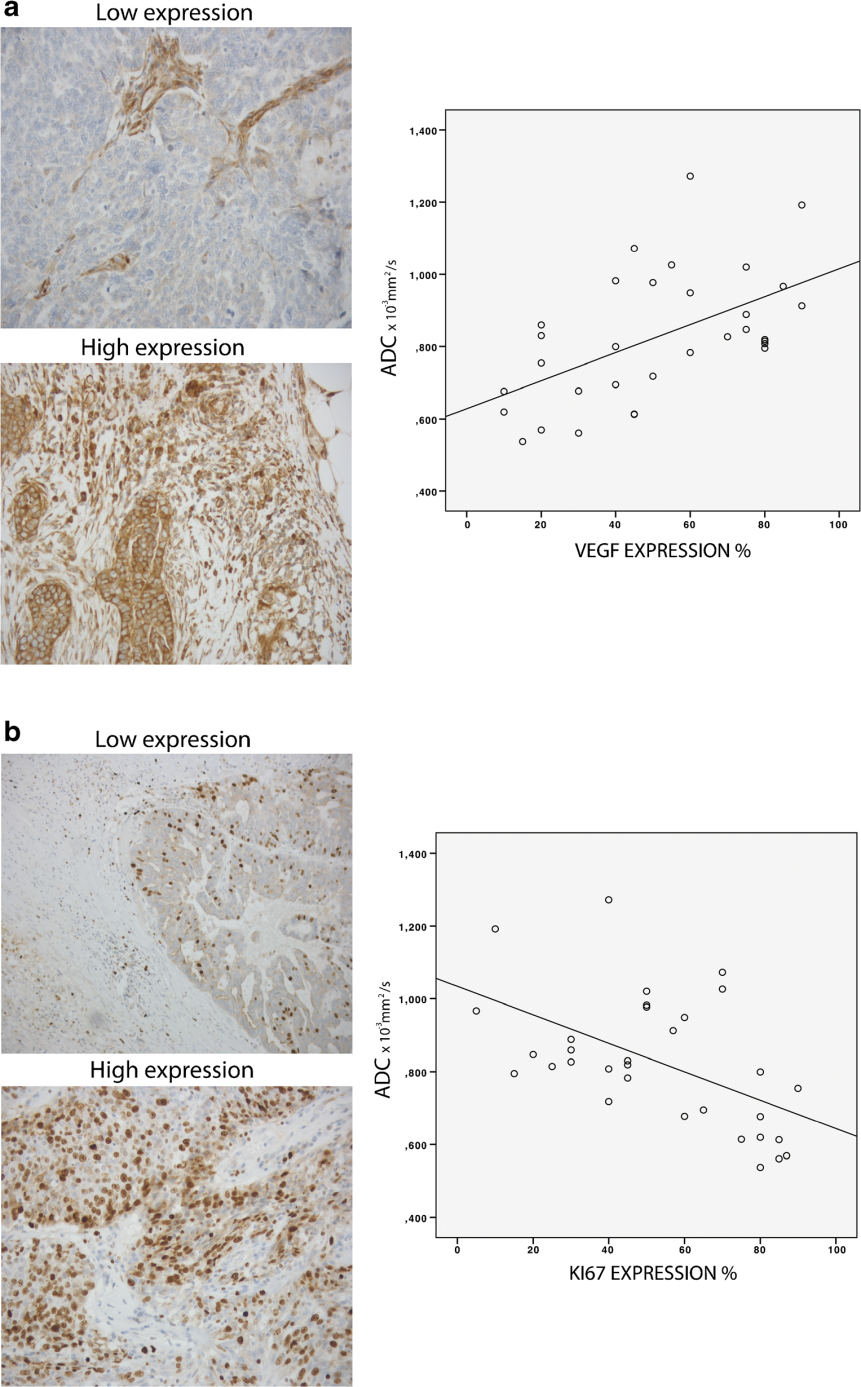
Histological samples of ovarian cancer tumours at 20x magnification and connection to apparent diffusion coefficients (ADCs). **a** Staining of vascular endothelial growth factor (VEGF) in epithelial cells with high and low expression. Scatter-dot graph illustrates the correlation between ADC when the ADC was measured using the whole lesion single plane region of interest (WLsp-ROI) and VEGF expression. **b** Ki-67 staining of the nucleus in high grade serous adenocarcinoma with high and low expression. Scatter-dot graph illustrates the correlation between ADC when the ADC was measured using the WLsp-ROI and Ki-67 expression

ADCs were significantly associated with VEGF protein expression in epithelial cells (WLsp-ROI *r* = 0.540, *P* = 0.001; S-ROI *r* = 0.552 *P* = 0.001) and inversely associated with Ki-67 protein expression in the nucleus (WLsp-ROI *r* = -0.540, *P* = 0.001; S-ROI *r* = -0.507, *P* = 0.003; Fig. 5), but not with other histopathologically measured variables in primary tumours. In metastases, ADCs inversely correlated with the mean lymphatic vessel size (WLsp-ROI: *r* = -0.618, *P* = 0.043).

### Correlation between ADCs and qRT-PCR analyses

In primary tumours, ADCs did not correlate with qRT-PCR variables. In metastases, ADCs (WLsp-ROI) significantly correlated with all VEGFRs mRNA levels (VEGFR-1: *r* = 0.836, *P* = 0.001; VEGFR-2: *r* = 0.764, *P* = 0.006; VEGFR-3: *r* = 0.627, *P* = 0.039). ADCs (WLsp-ROI) also significantly correlated with VEGF-C mRNA expression (*r* = 0.855, *P* = 0.001) but not with VEGF or VEGF-D mRNA expression.

### Recurrence-free survival

Thirty-six patients were included in the analysis of RFS. Eighteen patients experienced recurrence during the follow-up. The median RFS was 11 ± 6 months. ADCs did not have a significant effect on RFS in the Kaplan-Meier log rank test. In the univariate survival analysis, advanced stage, FIGO III-IV (*P* = 0.002), presence of residual tumour in operation (*P* = 0.008), presence of ascites (*P* = 0.036), non-sensitivity to platinum-based chemotherapy (*P* = 0.001), incomplete response to treatment (*P* = 0.006), and high Ki-67 expression (*P* = 0.037) were significant predictors of shorter RFS. None of these variables maintained their significance in the Cox multivariate analysis.

### Overall survival

**Fig. 6:**
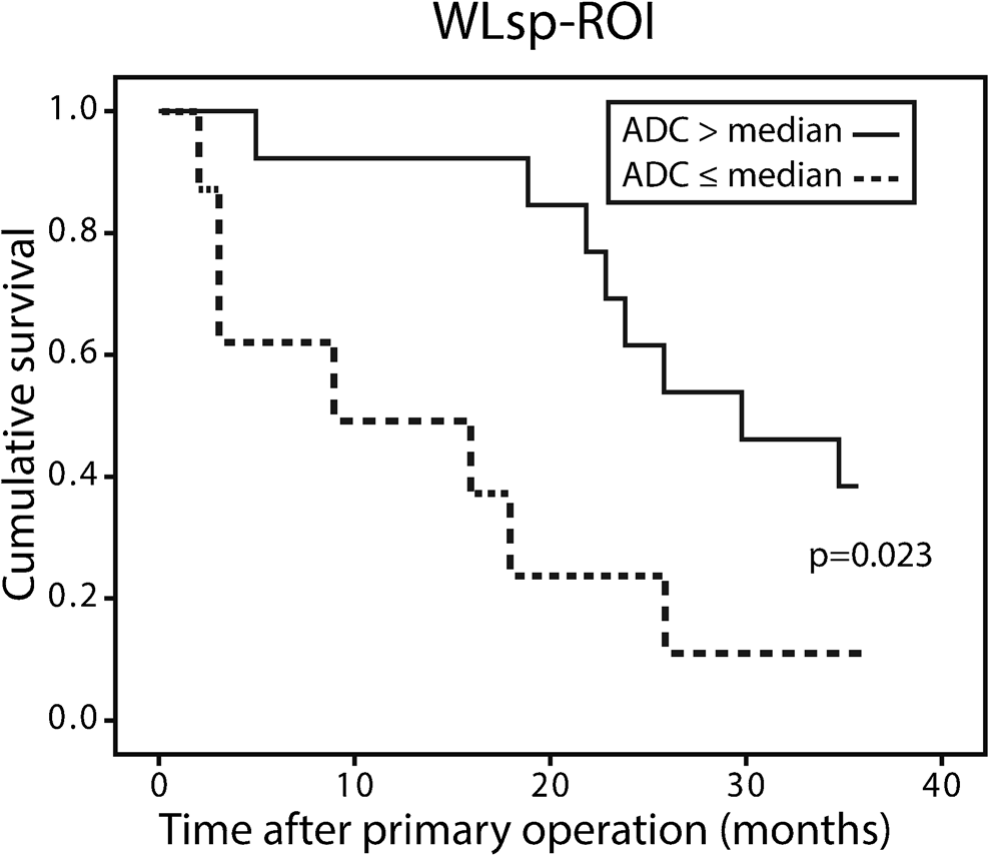
Univariate analysis of cumulative overall survival in relation to dichotomized apparent diffusion coefficients (ADCs). The 3-year overall survival was significantly prolonged in patients with high ADCs measured using the whole lesion single plane covered region of interest (WLsp-ROI)

The median follow-up time was 26 months (range 2-63 months, two patients having died two months after diagnosis). At the end of the follow-up, 16 (40%) patients with OC had died. The OS of the patients was 26 ± 12 months and the 3-year OS rate 38% (*n* = 26). During the 3-year follow-up (*n* = 26), lower ADCs predicted significantly poorer OS (WLsp-ROI *P* = 0.023, S-ROI *P* = 0.038) when assessing Kaplan-Meier curves by a log rank test (Fig. 6). In the univariate survival analysis, lower ADCs, the presence of residual tumour, an incomplete response to treatment, poor response to chemotherapy, and body mass index (BMI) > 25 kg/m^2^ were significant predictors of poorer OS. Bevacizumab treatment did not have prognostic significance in this patient cohort. In the Cox multivariate regression analysis, lower ADCs (*P* = 0.020), an incomplete response to treatment (*P* = 0.010), and BMI > 25 kg/m^2^ (*P* = 0.031) were independent predictors of poorer OS (Table 3).

**Table 3:** Univariate and multivariate analysis of 3-year overall survival

Variable	Univariate analysis	Multivariate analysis		
	*P*	Hazard ratio	95% CI	*P*
ADC for WLsp-ROI	0.023	10.204	(1.404-74.154)	0.020
Primary residual tumour (cut-off 1 cm)	0.007	0.497	(0.085-2.891)	n.s.
Response to treatment	<0.001			
complete response		0.760	(0.031-18.674)	0.008
partial response				n.s.
progressive disease		14.564	(1.912-110.950)	0.010
Platina sensitive	<0.001		.	n.s.
BMI (cut-off 25 kg/m^2^)	0.002	9.920	(1.200-81.950)	0.031

ADC = apparent diffusion coefficient, BMI = body mass index, CI = confidence interval, WLsp-ROI = whole lesion single plane covered region of interest, n.s. = not significant

## Discussion

We prospectively enrolled 40 patients with OC to study whether ADCs measured by 3.0T DWI imaging associated with histological severity of OC or predicted the clinical outcome. Our results illustrate that measurement of ADCs is a valuable tool for characterizing OC. In our cohort, reduced ADCs were associated with traditional histopathological prognostic markers, such as poorly differentiated tumours and high Ki-67 expression. ADCs also significantly correlated with VEGF protein expression in primary tumours epithelial cells and with VEGF receptor expression in metastases. Importantly, lower ADCs predicted significantly poorer OS at 3 years.

Analysis of ADCs has shown promise in increasing the precision of diagnosis, prognosis assessment, and predicting the therapeutic response in different cancers [13, 14, 17, 23], paralleling the results in preclinical studies [24]. In our cohort, ADCs measured with WLsp-ROI were lower in poorly differentiated primary tumours, an observation consistent with early studies [11, 18]. Grade is a significant predictor of OC outcome [2, 25]. Ki-67 is a nuclear protein associated with cellular proliferation, and high Ki-67 expression is associated with more aggressive disease [4]. In primary tumours, ADCs were inversely associated with Ki-67 protein expression measured with both WLsp- and S-ROI. Similar results have been published for prostate [18] and breast cancer [26].

ADC measurements were extracted from both larger ROIs covering the entire tumour at a single plane (WLsp-ROI) and defined subareas within tumours (S-ROI). ADCs measured from the entire tumour at a single plane were higher than the values with small subregions. In this cohort, ADC-values from S-ROIs proved to be inferior to WLsp-ROI in the prediction of OC histopathology and survival. Our results indicate that ADC values measured from WLsp-ROI are sufficient to be used as prognostic biomarkers in DWI-MRI of OC. The correlation of ADC measurements between two readers was excellent in primary tumours. In Bland-Altman analysis the 95% limits of agreement were slightly wider for S-ROI measurements in comparison to WLsp-ROI measurements (Fig. 2). The mean ADCs for the primary tumours were lower in our study than in many earlier studies [27, 28]. However, there are studies in which the ADCs are consistent with our results [11, 12, 16]. Conflicting ADCs in the literature could be caused by differences in ROI placement, scanners, diffusion gradients, the b values used and fitting of ADC data.

Interestingly, we observed a significant correlation between the ADCs measured with WLsp-ROI and 3-year OS in Cox regression analysis. However, there was no difference in 1- or 2-year survival. ADCs did not correlate with recurrence-free survival. There are no previous reports on the significance of ADC in the prediction of OC, but in cervical cancer lower ADCs significantly associate with worse survival [29].

In our cohort, higher ADCs were associated with high VEGF protein expression in endothelial cells. Previously, it has been shown that VEGF expression is high already in the early stage of disease; it is not growing exponentially when the tumour grows [30]. This could be a reason for the positive correlation between VEGF and ADC in our study. One previous study had shown that VEGF expression determined already in the early stage of disease showed prognostic value [31]. In a study of colorectal cancer, increased VEGF expression was associated with well-differentiated tumours [32].

VEGF expression has been shown to be higher in metastases than in primary tumours [33]. This is in line with our results; in this study VEGF protein expression was higher in metastases. The present study also shows that VEGF-C, VEGFR-1, -2, and -3 mRNA expression is higher in metastases than in related primary tumours. In a previous study, levels of VEGF-C, VEGF-D and VEGFR-3 proteins significantly increased in the presence of peritoneal metastases of OC outside the pelvis [34]. The presence of more mRNAs for angiogenic factors and their receptors may be expected when there is a need for accelerated neovascularization at metastatic sites. VEGF-C and its receptor VEGFR-3 are mediators of lymphangiogenesis [7], and higher expression in metastatic lesions compared to primary tumours reveals the possible role of lymphangiogenesis in metastatic tumour spread.

The strength of the present study is the prospectively collected OC cohort with DWI-MRI and multiple histopathological and angiogenesis markers analysed immunohistochemically and with qRT-PCR. Interobserver correlations of the analyses used were substantial. However, there are no consistent guidelines for DWI-MR imaging of patients with OC. The imaging protocol of the present study generated ADC maps from three b values (including b = 0). Intravoxel incoherent motion (IVIM) is an imaging technique that makes separate estimations of tissue perfusion and diffusivity using multi-b-value DWI. Recently, implementation of IVIM with DWI has been studied, for example, in patients with cervical cancer [35] and breast cancer [36] and has shown promise in improving the specificity of MRI. Unfortunately, our imaging protocol did not contain IVIM parameters. Additional limitations were the small study population and heterogeneous histological types of epithelial OC. However, in clinical situations, the cancer histology is not known pre-operatively, and it would be very beneficial if ADCs were useful in all histological types. We had to exclude four patients due to technical reasons in DWI-MRI and five from histological and qRT-PCR analyses due to neoadjuvant chemotherapy, which may have affected our results.

DWI, which is easily incorporated into standard MRI protocols, is a new promising tool for the diagnosis and follow-up of OC patients. In our cohort, there was a correlation between ADCs and histopathological prognostic markers and outcome. Our results indicate that WLsp-ROI can reproducibly be used to measure ADC values, and that it can be used as a prognostic biomarker in OC. Larger scale studies are needed to confirm our observations and to clarify the prognostic value of DWI in patients with OC.

## Abbreviations

OC: Ovarian cancer
DWI: Diffusion-weighted magnetic resonance imaging
ROI: Regions of interest
S-ROI: Five small ROIs
WLsp-ROI: Whole lesion single plane region of interest
ADC: Apparent diffusion coefficient
qRT-PCR: Quantitative reverse transcription polymerase chain reaction
VEGF: Vascular endothelial growth factor
VEGFR: Vascular endothelial growth factor receptor
CT: Computed tomography
FIGO: The International Federation of Gynecology and Obstetrics
WHO: The World Health Organization
ICC: Interclass correlation coefficient
TR: Repetition time
TE: Echo time
FFE: Fast field echo
SPAIR: Spectral attenuated inversion recovery
DWIBS: Diffusion-weighted imaging with background body signal suppression
PPIA: Peptidylprolyl isomerase A
OS: Overall survival
RFS: Recurrence-free survival
IVIM: Intravoxel incoherent motion
